# Mississippi Medical Monthly

**Published:** 1891-11

**Authors:** 


WE CALL ATTENTION to the advertisement in this issue of the *Mississippi Medical Monthly*, edited and published at Meridian, Miss., by N. L. Clarke, M. D., and H. H. Harralson, M. D. These gentlemen stand at the head of the profession of their State. The former is President of the Lauderdale County Medical Association and one of the Vice-Presidents of the Mississippi State Medical Association. The latter is the present Secretary of the Mississippi State Medical Association, and has read several valuable articles before that body. Both are successful practitioners of medicine, and their journal is a rich addition to the medical literature of the South.

